# Supplementary material for: A fault-intrusion-tolerant system and deadline-aware algorithm for scheduling scientific workflow in the cloud
Source: PeerJ Comput Sci. 2021 Nov 2;7:e747. doi: 10.7717/peerj-cs.747 (PMC8576554; doi:10.7717/peerj-cs.747)
Supplement: Supplemental Information 6 [file peerj-cs-07-747-s006.zip › Myproject1.1/docs/org/cloudbus/cloudsim/Vm.html]

Vm


JavaScript is disabled on your browser.


- Overview
- Package
- Class
- Use
- Tree
- Deprecated
- Index
- Help

- Prev Class
- Next Class

- Frames
- No Frames

- All Classes

- Summary:
- Nested |
- Field |
- Constr |
- Method

- Detail:
- Field |
- Constr |
- Method


org.cloudbus.cloudsim

## Class Vm

- java.lang.Object
- - org.cloudbus.cloudsim.Vm

- Direct Known Subclasses:
  :   CondorVM, NetworkVm, PowerVm

  ---

    

  ```
  public class Vm
  extends java.lang.Object
  ```

  Vm represents a VM: it runs inside a Host, sharing hostList with other VMs. It processes
  cloudlets. This processing happens according to a policy, defined by the CloudletScheduler. Each
  VM has a owner, which can submit cloudlets to the VM to be executed

  Since:
  :   CloudSim Toolkit 1.0

- - ### Constructor Summary

    Constructors

    Constructor and Description

    `Vm(int id,
    int userId,
    double mips,
    int numberOfPes,
    int ram,
    long bw,
    long size,
    java.lang.String vmm,
    CloudletScheduler cloudletScheduler)`

    Creates a new VMCharacteristics object.
  - ### Method Summary

    Methods

    | Modifier and Type | Method and Description |
    | --- | --- |
    | `void` | `addStateHistoryEntry(double time, double allocatedMips, double requestedMips, boolean isInMigration)` Adds the state history entry. |
    | `long` | `getBw()` Gets the amount of bandwidth. |
    | `CloudletScheduler` | `getCloudletScheduler()` Gets the vm scheduler. |
    | `long` | `getCurrentAllocatedBw()` Gets the current allocated bw. |
    | `java.util.List<java.lang.Double>` | `getCurrentAllocatedMips()` Gets the current allocated mips. |
    | `int` | `getCurrentAllocatedRam()` Gets the current allocated ram. |
    | `long` | `getCurrentAllocatedSize()` Gets the current allocated size. |
    | `long` | `getCurrentRequestedBw()` Gets the current requested bw. |
    | `double` | `getCurrentRequestedMaxMips()` Gets the current requested max mips among all virtual PEs. |
    | `java.util.List<java.lang.Double>` | `getCurrentRequestedMips()` Gets the current requested mips. |
    | `int` | `getCurrentRequestedRam()` Gets the current requested ram. |
    | `double` | `getCurrentRequestedTotalMips()` Gets the current requested total mips. |
    | `Host` | `getHost()` Gets the host. |
    | `int` | `getId()` Gets the id. |
    | `double` | `getMips()` Gets the mips. |
    | `int` | `getNumberOfPes()` Gets the number of pes. |
    | `int` | `getRam()` Gets the amount of ram. |
    | `long` | `getSize()` Gets the amount of storage. |
    | `java.util.List<VmStateHistoryEntry>` | `getStateHistory()` Gets the state history. |
    | `double` | `getTotalUtilizationOfCpu(double time)` Get utilization created by all clouddlets running on this VM. |
    | `double` | `getTotalUtilizationOfCpuMips(double time)` Get utilization created by all cloudlets running on this VM in MIPS. |
    | `java.lang.String` | `getUid()` Get unique string identificator of the VM. |
    | `static java.lang.String` | `getUid(int userId, int vmId)` Generate unique string identificator of the VM. |
    | `int` | `getUserId()` Gets the ID of the owner of the VM. |
    | `java.lang.String` | `getVmm()` Gets the VMM. |
    | `boolean` | `isBeingInstantiated()` Checks if is being instantiated. |
    | `boolean` | `isInMigration()` Checks if is in migration. |
    | `void` | `setBeingInstantiated(boolean beingInstantiated)` Sets the being instantiated. |
    | `void` | `setBw(long bw)` Sets the amount of bandwidth. |
    | `protected void` | `setCloudletScheduler(CloudletScheduler cloudletScheduler)` Sets the vm scheduler. |
    | `void` | `setCurrentAllocatedBw(long currentAllocatedBw)` Sets the current allocated bw. |
    | `void` | `setCurrentAllocatedMips(java.util.List<java.lang.Double> currentAllocatedMips)` Sets the current allocated mips. |
    | `void` | `setCurrentAllocatedRam(int currentAllocatedRam)` Sets the current allocated ram. |
    | `protected void` | `setCurrentAllocatedSize(long currentAllocatedSize)` Sets the current allocated size. |
    | `void` | `setHost(Host host)` Sets the host that runs this VM. |
    | `protected void` | `setId(int id)` Sets the id. |
    | `void` | `setInMigration(boolean inMigration)` Sets the in migration. |
    | `protected void` | `setMips(double mips)` Sets the mips. |
    | `protected void` | `setNumberOfPes(int numberOfPes)` Sets the number of pes. |
    | `void` | `setRam(int ram)` Sets the amount of ram. |
    | `void` | `setSize(long size)` Sets the amount of storage. |
    | `void` | `setUid(java.lang.String uid)` Sets the uid. |
    | `protected void` | `setUserId(int userId)` Sets the user id. |
    | `protected void` | `setVmm(java.lang.String vmm)` Sets the VMM. |
    | `double` | `updateVmProcessing(double currentTime, java.util.List<java.lang.Double> mipsShare)` Updates the processing of cloudlets running on this VM. |

    - ### Methods inherited from class java.lang.Object

      `clone, equals, finalize, getClass, hashCode, notify, notifyAll, toString, wait, wait, wait`

- - ### Constructor Detail


    - #### Vm

      ```
      public Vm(int id,
        int userId,
        double mips,
        int numberOfPes,
        int ram,
        long bw,
        long size,
        java.lang.String vmm,
        CloudletScheduler cloudletScheduler)
      ```

      Creates a new VMCharacteristics object.

      Parameters:
      :   `id` - unique ID of the VM
      :   `userId` - ID of the VM's owner
      :   `mips` - the mips
      :   `numberOfPes` - amount of CPUs
      :   `ram` - amount of ram
      :   `bw` - amount of bandwidth
      :   `size` - amount of storage
      :   `vmm` - virtual machine monitor
      :   `cloudletScheduler` - cloudletScheduler policy for cloudlets
  - ### Method Detail


    - #### updateVmProcessing

      ```
      public double updateVmProcessing(double currentTime,
                              java.util.List<java.lang.Double> mipsShare)
      ```

      Updates the processing of cloudlets running on this VM.

      Parameters:
      :   `currentTime` - current simulation time
      :   `mipsShare` - array with MIPS share of each Pe available to the scheduler

      Returns:
      :   time predicted completion time of the earliest finishing cloudlet, or 0 if there is no
          next events


    - #### getCurrentRequestedMips

      ```
      public java.util.List<java.lang.Double> getCurrentRequestedMips()
      ```

      Gets the current requested mips.

      Returns:
      :   the current requested mips


    - #### getCurrentRequestedTotalMips

      ```
      public double getCurrentRequestedTotalMips()
      ```

      Gets the current requested total mips.

      Returns:
      :   the current requested total mips


    - #### getCurrentRequestedMaxMips

      ```
      public double getCurrentRequestedMaxMips()
      ```

      Gets the current requested max mips among all virtual PEs.

      Returns:
      :   the current requested max mips


    - #### getCurrentRequestedBw

      ```
      public long getCurrentRequestedBw()
      ```

      Gets the current requested bw.

      Returns:
      :   the current requested bw


    - #### getCurrentRequestedRam

      ```
      public int getCurrentRequestedRam()
      ```

      Gets the current requested ram.

      Returns:
      :   the current requested ram


    - #### getTotalUtilizationOfCpu

      ```
      public double getTotalUtilizationOfCpu(double time)
      ```

      Get utilization created by all clouddlets running on this VM.

      Parameters:
      :   `time` - the time

      Returns:
      :   total utilization


    - #### getTotalUtilizationOfCpuMips

      ```
      public double getTotalUtilizationOfCpuMips(double time)
      ```

      Get utilization created by all cloudlets running on this VM in MIPS.

      Parameters:
      :   `time` - the time

      Returns:
      :   total utilization


    - #### setUid

      ```
      public void setUid(java.lang.String uid)
      ```

      Sets the uid.

      Parameters:
      :   `uid` - the new uid


    - #### getUid

      ```
      public java.lang.String getUid()
      ```

      Get unique string identificator of the VM.

      Returns:
      :   string uid


    - #### getUid

      ```
      public static java.lang.String getUid(int userId,
                            int vmId)
      ```

      Generate unique string identificator of the VM.

      Parameters:
      :   `userId` - the user id
      :   `vmId` - the vm id

      Returns:
      :   string uid


    - #### getId

      ```
      public int getId()
      ```

      Gets the id.

      Returns:
      :   the id


    - #### setId

      ```
      protected void setId(int id)
      ```

      Sets the id.

      Parameters:
      :   `id` - the new id


    - #### setUserId

      ```
      protected void setUserId(int userId)
      ```

      Sets the user id.

      Parameters:
      :   `userId` - the new user id


    - #### getUserId

      ```
      public int getUserId()
      ```

      Gets the ID of the owner of the VM.

      Returns:
      :   VM's owner ID


    - #### getMips

      ```
      public double getMips()
      ```

      Gets the mips.

      Returns:
      :   the mips


    - #### setMips

      ```
      protected void setMips(double mips)
      ```

      Sets the mips.

      Parameters:
      :   `mips` - the new mips


    - #### getNumberOfPes

      ```
      public int getNumberOfPes()
      ```

      Gets the number of pes.

      Returns:
      :   the number of pes


    - #### setNumberOfPes

      ```
      protected void setNumberOfPes(int numberOfPes)
      ```

      Sets the number of pes.

      Parameters:
      :   `numberOfPes` - the new number of pes


    - #### getRam

      ```
      public int getRam()
      ```

      Gets the amount of ram.

      Returns:
      :   amount of ram


    - #### setRam

      ```
      public void setRam(int ram)
      ```

      Sets the amount of ram.

      Parameters:
      :   `ram` - new amount of ram


    - #### getBw

      ```
      public long getBw()
      ```

      Gets the amount of bandwidth.

      Returns:
      :   amount of bandwidth


    - #### setBw

      ```
      public void setBw(long bw)
      ```

      Sets the amount of bandwidth.

      Parameters:
      :   `bw` - new amount of bandwidth


    - #### getSize

      ```
      public long getSize()
      ```

      Gets the amount of storage.

      Returns:
      :   amount of storage


    - #### setSize

      ```
      public void setSize(long size)
      ```

      Sets the amount of storage.

      Parameters:
      :   `size` - new amount of storage


    - #### getVmm

      ```
      public java.lang.String getVmm()
      ```

      Gets the VMM.

      Returns:
      :   VMM


    - #### setVmm

      ```
      protected void setVmm(java.lang.String vmm)
      ```

      Sets the VMM.

      Parameters:
      :   `vmm` - the new VMM


    - #### setHost

      ```
      public void setHost(Host host)
      ```

      Sets the host that runs this VM.

      Parameters:
      :   `host` - Host running the VM


    - #### getHost

      ```
      public Host getHost()
      ```

      Gets the host.

      Returns:
      :   the host


    - #### getCloudletScheduler

      ```
      public CloudletScheduler getCloudletScheduler()
      ```

      Gets the vm scheduler.

      Returns:
      :   the vm scheduler


    - #### setCloudletScheduler

      ```
      protected void setCloudletScheduler(CloudletScheduler cloudletScheduler)
      ```

      Sets the vm scheduler.

      Parameters:
      :   `cloudletScheduler` - the new vm scheduler


    - #### isInMigration

      ```
      public boolean isInMigration()
      ```

      Checks if is in migration.

      Returns:
      :   true, if is in migration


    - #### setInMigration

      ```
      public void setInMigration(boolean inMigration)
      ```

      Sets the in migration.

      Parameters:
      :   `inMigration` - the new in migration


    - #### getCurrentAllocatedSize

      ```
      public long getCurrentAllocatedSize()
      ```

      Gets the current allocated size.

      Returns:
      :   the current allocated size


    - #### setCurrentAllocatedSize

      ```
      protected void setCurrentAllocatedSize(long currentAllocatedSize)
      ```

      Sets the current allocated size.

      Parameters:
      :   `currentAllocatedSize` - the new current allocated size


    - #### getCurrentAllocatedRam

      ```
      public int getCurrentAllocatedRam()
      ```

      Gets the current allocated ram.

      Returns:
      :   the current allocated ram


    - #### setCurrentAllocatedRam

      ```
      public void setCurrentAllocatedRam(int currentAllocatedRam)
      ```

      Sets the current allocated ram.

      Parameters:
      :   `currentAllocatedRam` - the new current allocated ram


    - #### getCurrentAllocatedBw

      ```
      public long getCurrentAllocatedBw()
      ```

      Gets the current allocated bw.

      Returns:
      :   the current allocated bw


    - #### setCurrentAllocatedBw

      ```
      public void setCurrentAllocatedBw(long currentAllocatedBw)
      ```

      Sets the current allocated bw.

      Parameters:
      :   `currentAllocatedBw` - the new current allocated bw


    - #### getCurrentAllocatedMips

      ```
      public java.util.List<java.lang.Double> getCurrentAllocatedMips()
      ```

      Gets the current allocated mips.

      Returns:
      :   the current allocated mips


    - #### setCurrentAllocatedMips

      ```
      public void setCurrentAllocatedMips(java.util.List<java.lang.Double> currentAllocatedMips)
      ```

      Sets the current allocated mips.

      Parameters:
      :   `currentAllocatedMips` - the new current allocated mips


    - #### isBeingInstantiated

      ```
      public boolean isBeingInstantiated()
      ```

      Checks if is being instantiated.

      Returns:
      :   true, if is being instantiated


    - #### setBeingInstantiated

      ```
      public void setBeingInstantiated(boolean beingInstantiated)
      ```

      Sets the being instantiated.

      Parameters:
      :   `beingInstantiated` - the new being instantiated


    - #### getStateHistory

      ```
      public java.util.List<VmStateHistoryEntry> getStateHistory()
      ```

      Gets the state history.

      Returns:
      :   the state history


    - #### addStateHistoryEntry

      ```
      public void addStateHistoryEntry(double time,
                              double allocatedMips,
                              double requestedMips,
                              boolean isInMigration)
      ```

      Adds the state history entry.

      Parameters:
      :   `time` - the time
      :   `allocatedMips` - the allocated mips
      :   `requestedMips` - the requested mips
      :   `isInMigration` - the is in migration


- Overview
- Package
- Class
- Use
- Tree
- Deprecated
- Index
- Help

- Prev Class
- Next Class

- Frames
- No Frames

- All Classes

- Summary:
- Nested |
- Field |
- Constr |
- Method

- Detail:
- Field |
- Constr |
- Method
